# A Novel 2-Methoxyestradiol Derivative: Disrupting Mitosis Inhibiting Cell Motility and Inducing Apoptosis in HeLa Cells In Vitro

**DOI:** 10.3390/pharmaceutics16050622

**Published:** 2024-05-06

**Authors:** Isaac Kinyua Njangiru, Noémi Bózsity-Faragó, Vivien Erzsébet Resch, Gábor Paragi, Éva Frank, György T. Balogh, István Zupkó, Renáta Minorics

**Affiliations:** 1 Institute of Pharmacodynamics and Biopharmacy, University of Szeged, Eötvös u. 6, H-6720 Szeged, Hungarybozsity-farago.noemi@szte.hu (N.B.-F.);; 2 Department of Medicinal Chemistry, University of Szeged, Dóm tér 8, H-6720 Szeged, Hungary; 3 Department of Theoretical Physics, University of Szeged, Tisza Lajos krt. 84-86, 6720 Szeged, Hungary; 4 Institute of Physics, University of Pécs, H-7622 Pécs, Hungary; 5 Department of Molecular and Analytical Chemistry, University of Szeged, Dóm tér 7-8, H-6720 Szeged, Hungary; 6 Department of Pharmaceutical Chemistry, Semmelweis University, Hőgyes Endre Street 7-9, H-1092 Budapest, Hungary

**Keywords:** anticancer, metastasis, apoptosis, cell cycle, tubulin, molecular docking

## Abstract

The clinical application of 2-methoxyestradiol (2ME) in cancer therapy has been limited by its low solubility and rapid metabolism. Derivatives of 2ME have been synthesised to enhance bioavailability and decrease hepatic metabolism. Compound **4a**, an analog of 2ME, has demonstrated exceptional pharmacological activity, in addition to promising pharmacokinetic profile. Our study, therefore, aimed at exploring the anticancer effects of **4a** on the cervical cancer cell line, HeLa. Compound **4a** exhibited a significant and dose-dependent antimetastatic and antiinvasive impact on HeLa cells, as determined by wound-healing and Boyden chamber assays, respectively. Hoechst/Propidium iodide (HOPI) double staining showcased a substantial induction of apoptosis via **4a**, with minimal necrotic effect. Flow cytometry revealed a significant G2/M phase arrest, accompanied by a noteworthy rise in the sub-G1 cell population, indicating apoptosis, 18 h post-treatment. Moreover, a cell-independent tubulin polymerisation assay illustrated compound **4a**’s ability to stabilise microtubules by promoting tubulin polymerisation. Molecular modelling experiments depicted that **4a** interacts with the colchicine-binding site, nestled between the α and β tubulin dimers. Furthermore, **4a** displayed an affinity for binding to and activating ER-α, as demonstrated by the luciferase reporter assay. These findings underscore the potential of **4a** in inhibiting HPV18+ cervical cancer proliferation and cellular motility.

## 1. Introduction

Oestrogens, synthesised from cholesterol within the human body, play a vital role as hormones in the female reproductive system [1]. These hormones have extensive interactions with multiple organ systems and significantly influence various physiological events in women. However, an excess of these hormones can cause abnormal growth in hormone-sensitive cells. This elevated expression heightens the likelihood of developing hormone-dependent cancers like breast, uterine, ovarian, and endometrial cancers [2]. Cervical cancer is the fourth most common cancer in women worldwide, responsible for one-fifth of cancer-related deaths [3]. Infection with oncogenic strains of the human papillomavirus (HPV) leads to the production of E6 and E7 proteins, which inhibit the function of crucial tumour suppressor genes, notably p53 and Retinoblastoma (Rb), respectively [4]. Unfortunately, long-term survival rates for individuals with International Federation of Obstetrics and Gynaecology (FIGO stage) IIIA–C1 disease remain at approximately 50% [5], and prognoses are even worse for patients with para-aortic lymph node involvement (stage IIIC2) of cervical cancer [6]. Even though cervical cancer rates have globally decreased over the past decade due to enhanced screening and the introduction of the HPV vaccine, it remains a significant health concern, especially in developing countries [7].

Nearly 85% of new cases worldwide occur in less developed regions, often diagnosed at advanced stages of malignancy, severely impacting the chances of successful treatment [8].Tragically, approximately nine out of ten cervical cancer deaths occur in these areas [9]. The southern African region is particularly at high risk, with an incidence rate of 31.5 women per 100,000 [10]. This global disparity in the disease burden can be attributed to limited healthcare resources in developing countries, highlighting the potential for improving treatment, especially at advanced stages of malignancy [11]. The available treatment options for cervical cancer currently include surgery, radiation therapy, and chemotherapy [12]. Chemotherapy is effective since it targets rapidly dividing cancer cells, which are more vulnerable to drugs used in this therapy [13]. Many of the existing chemotherapeutic drugs focus on disrupting the cell cycle, with the aim of halting cancer cells in the phase of cell division called mitosis. This inhibition of hyperproliferation eventually leads to programmed cell death [14]. Among these drugs, those that specifically target microtubules and interfere with the functioning of the mitotic spindle have proven to be highly successful [15,16]. These microtubule-targeting agents (MTAs) disrupt the normal dynamics of spindle microtubules, preventing cells from passing though the spindle checkpoint and entering anaphase. As a result, cells become blocked in the metaphase stage of mitosis [14]. Prolonged treatment with MTAs leads to a continuous arrest in mitosis, eventually causing an abnormal exit from mitosis and triggering cell death [17]. 

Extensive research has focused on chemical modifications of the phenolic A-ring at the C-2 position and the five-membered D-ring at the C-17 position in natural steroids, such as estrone (E) and oestradiol (E2), for drug design purposes. Derivatives based on C-2-substituted E or E2 have garnered significant interest due to their demonstrated ability to exhibit reduced or no binding to the oestrogen receptor (ER), thereby lacking hormonal activity [18]. Moreover, these compounds, along with their analogs modified at the C-17 position, have exhibited noteworthy antiproliferative activity against various human cancer cell lines [19]. One prominent example is 2-methoxyestradiol (2ME), an endogenous metabolite of E2, known for its antiproliferative, antimitotic, and pro-apoptotic properties, both in vitro and in vivo [20,21]. It exerts its inhibitory effects on cancer cell proliferation by targeting the microtubule network, independent of the oestrogen receptor status [22].

Marketed as Panzem by Entremed Inc., 2ME has successfully completed both phase I and II clinical trials for the treatment of multiple myeloma, glioblastoma, metastatic breast cancer, prostate and ovarian cancers, as well as several other solid malignancies [23]. Despite its promises, 2ME has been reported to have limited bioavailability, occasioned by poor solubility, and rapid hepatic metabolism due to the conjugation and oxidation [24] of the hydroxyl groups (-OH) at C-3 and C-17. Researchers are currently striving to harness the beneficial attributes of 2ME in order to create innovative therapeutic analogs that offer enhanced potency and safety.

These analogs aim to exhibit superior tumour selectivity and specificity, improved bioavailability, and reduced hepatic metabolism. In our previous research study [19], we comprehensively analysed the pharmacokinetics and anti-proliferative potential of a set of estrone, oestradiol, and 17β benzylamino derivatives. These modifications were directed at C-2 and C-17 positions and were tested on human adherent cervical (HeLa), ovarian (A2780), and breast (MDA-MB-231) carcinoma cell lines. Among the analogs studied, 2-[(dimethylamino)methyl]-oestradiol, designated as **4a**, demonstrated significant improvement in addressing certain pharmacokinetic weaknesses associated with 2ME. Additionally, we observed the growth inhibitory effect of compound **4a** on various human adherent gynecologically derived cancer cell lines, as well as on non-cancerous mouse fibroblast (NIH/3T3) cells [19]. Furthermore, **4a** was found to be the most effective analog against the HeLa cell line, with an IC_50_ of 4.53 μM. However, evaluating the anticancer potential of a compound solely based on its effects on malignant cell lines or animal models, without considering the selectivity index (SI), is insufficient for guiding subsequent clinical research [25]. Therefore, a criterion of an SI value of ≥10 is utilised to identify promising hit molecules which deserve further investigation [26]. Based on the ratio of IC_50_ values from our most susceptible cancerous cell line HeLa and non-cancerous mouse fibroblast (NIH/3T3; 70.44 μM) cells, compound **4a** exhibits a selectivity index of 15.6, surpassing the established threshold. Since the SI is calculated from in vitro results generated from cell-based experiments, it cannot substitute a detailed toxicological evaluation, which requires further experimentation. However, the promising selectivity of **4a** justified additional investigations.

Our present study, therefore, aims at elucidating how compound **4a** exerts its anti-proliferative effects on the cervical cancer cell line, HeLa. We will investigate its ability to inhibit cancer metastasis using wound-healing assays, while Boyden chamber assays will assess its anti-invasive properties. We will examine its potential to induce cellular apoptosis and necrosis using Hoechst/Propidium iodide staining and examine its impact on cell cycle progression through flow cytometry. Its influence on microtubule dynamics will be evaluated using tubulin polymerisation assays, while molecular docking will unfold how **4a** interacts with the tubulin-binding sites. Additionally, we will assess its oestrogenic-like activity via luciferase reporter assays.

## 2. Materials and Methods

### 2.1. Chemical Structure

**Figure 1:**
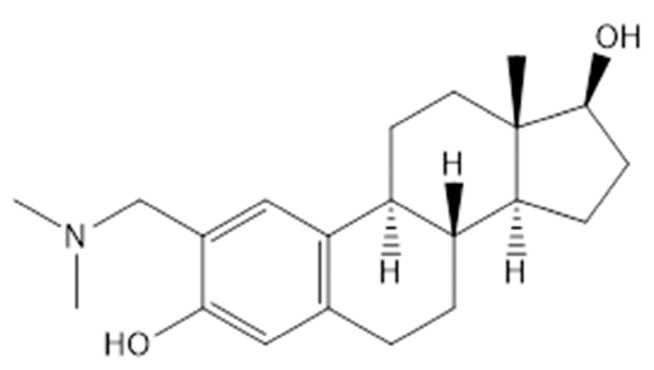
The chemical structure of the tested compound (**4a**).

The synthesis and chemical characterisation of **4a** (2-[(dimethylamino)methyl]-oestradiol), was carried out as reported previously [19]. The chemical structure of **4a** is presented in Figure 1.

### 2.2. Cell Lines

The HeLa cell line, derived from human cervical carcinoma and positive for HPV-18 and the ERα positive breast cancer cell lines MCF7 and T47D, were obtained from the ECACC (European Collection of Authenticated Cell Cultures, Salisbury, UK) and utilised with passage number < 20 generations. The cells were cultured in Eagle’s Minimal Essential Medium (EMEM), supplemented with 10% foetal bovine serum (FBS), 1% non-essential amino acid (NEAA), and a mixture of 1% penicillin, streptomycin, and amphotericin B. Notably, the media used in the maintenance of T47D cells was additionally supplemented with 1% L-glutamine. The culture was maintained at 37 °C in a 5% humidified carbon dioxide (CO_2_) atmosphere. All media, supplements, chemicals, and kits used in the experiments, unless specified otherwise, were purchased from Capricorn Scientific Ltd. (Ebsdorfergrund, Germany).

### 2.3. Hoechst 33258–Propidium Iodide Fluorescent Double Staining

Fluorescence staining was conducted to observe the morphological changes induced by the apoptotic process in cells treated with the compound **4a**. HeLa cell suspension was seeded into 6-well plates at a density of 150,000 cells per well and allowed to repose for 24 h before exposure to different concentrations of our test substance (1.0, 2.5, 5.0, and 10 μM) for 48 h. To distinguish apoptotic and necrotic cell populations, the cells were stained with lipophilic Hoechst 33258 (1 μg/mL, HO) and hydrophilic propidium iodide (3 μg/mL, PI) for 90 min at 37 °C in a CO_2_ incubator. Following the removal of the remaining dye-containing medium, a Nikon Eclipse TS100 fluorescence microscope (Nikon Instruments Europe, Amstelveen, The Netherlands) was used to capture at least 4 images per condition. The microscope was equipped with suitable optical blocks for Hoechst 33258 (excitation: 360/40 nm bandpass filter, emission: 460/50 nm bandpass filter, and 400 nm dichromatic mirror) and propidium iodide (excitation: 500/20 nm bandpass filter, emission: 520 nm long pass filter, and 515 nm dichromatic mirror). This technique enables differentiation between early apoptosis and secondary necrosis by examining the nuclear morphology and membrane integrity of cells. The HO dye solution readily permeates into the nuclei of all cells, resulting in a uniform blue staining in the nuclei of the viable cells. However, during the apoptotic process, distinct signs of bright chromatin condensation and nuclear fragmentation become evident. The uptake of PI indicates a compromised membrane integrity, and, in the case of late apoptosis or necrosis, the cell nuclei exhibit a red staining.

### 2.4. Cell Cycle Analysis Using Flow Cytometry

Cell cycle analysis was performed to elucidate a possible mechanism of action of the compound **4a**. Cells were seeded in a 12-well plate at a density of 120,000 cells per well and incubated overnight. Afterwards, the cells were treated with 1 mL of fresh EMEM media containing the desired concentration of **4a** and incubated for 48 h. The supernatant from each treatment was collected separately, and the cells were washed with PBS and detached using trypsin. The resulting supernatant containing the cells was centrifuged at 1300 rpm for 5 min. The cells were then washed with PBS and fixed with 70% ice-cold ethanol at −20 °C for 20 min. To stain the DNA content, the cells were treated with 300 µL of staining solution, which consisted of 10 µg/mL PI, 0.1% sodium citrate, 0.1% triton-X, and 10 µg/mL RNase-A, all dissolved in injectable water. The staining process was carried out in the dark for 30 min. The cells were subjected to analysis using a flow cytometer (CytoFLEX-V0-B4-RO, Beckman Coulter, Brea, CA, USA) with a minimum of 10,000 events per sample being evaluated during each analysis. The data obtained were processed using ModFit LT 3.3.11 software (Verity Software House, Topsham, ME, USA). Untreated cells were utilised as the control group. Apoptotic cells were identified as the hypodiploid (subG1) cell population. The cell cycle analysis experiments were conducted twice, with three parallel samples for each condition.

### 2.5. MTT Assay for Cell Viability

The extent of the inhibition of cell proliferation by the test compound was determined using the MTT (3-(4,5-dimethylthiazol-2-yl)-2,5-diphenyltetrazolium bromide) assay. Exponentially growing HeLa cells were seeded into 96-well plates at a density of 1.0 × 10^4^ cells per well in 100 µL of media and incubated for 24 h to facilitate cell attachment. Subsequently, the cells were treated with test compounds formulated in 100 µL of media at six different concentrations of compounds ranging, from 0.1 to 30 µmol. The control group received only media treatment. After 24 h of incubation, 22 µL of MTT solution (5 mg/mL in PBS; Duchefa Biochemie BV, Haarlem, The Netherlands) was added and incubated for 4 h at 37 °C in a humidified 5% CO_2_ environment. The medium was subsequently aspirated, and 100 µL of DMSO was added to dissolve the formazan crystals produced by the intact cell’s mitochondrial enzymes. The mixture was then mechanically shaken for 30 min. Absorbance measurements were taken at 545 nm using a microplate UV-VIS reader (SPECTRO star Nano, BMG Labtech GmbH, Offenburg, Germany), followed by the calculation of the percentage cell viability using the GraphPad Prism 5.01 (GraphPad Software, San Diego, CA, USA). All in vitro experiments were performed twice with five replicates. Stock solutions of the tested substances (10 mM) were prepared in DMSO. The highest DMSO content in the medium (0.3%) did not significantly affect cell proliferation.

### 2.6. Tubulin Polymerisation Assay

To assess the impact of test compound **4a** on the microtubular system, a cell-free tubulin polymerisation assay was conducted using a commercially available kit (Cytoskeleton Inc., Denver, CO, USA), largely in accordance with manufacturer’s instructions, though with some modifications. The assay was performed in a pre-heated 96-well plate, with each condition tested in two parallel wells. Two wells were assigned as the experimental control and received 10 µL of general tubulin buffer (GTB), while the next two wells were assigned as the positive control and received a similar volume of 10 µM paclitaxel. Various concentrations of the test compound (50, 150, 300, and 600 µM) were added in similar volumes, and the polymerisation reaction was initiated by adding 100 µL of tubulin to each well. Absorbance readings were measured every minute for 60 min at 340 nm, following a kinetic measurement protocol. A similar experiment was repeated with the absence of glycerol in the reaction mixture and in exclusion of 50, 150 µM of our test compound. A polymerisation curve was fitted to the collected data to demonstrate changes in the tubulin polymerisation caused by the test compound. The maximum rate of tubulin polymerisation (Vmax) was determined as the highest difference between a set of three absorbance values at any two consecutive intervals.

### 2.7. Wound-Healing Assay

Due to the significant contribution of cell motility to tumour metastasis, we performed a wound-healing assay, utilizing a 2D model, to explore the antimigratory potential of compound **4a** on HeLa cells. Special silicon inserts (Ibidi GmbH, Gräfelfing, Germany) were utilised, with 50,000 cells per well implanted on 12-well plates in a standard EMEM medium supplemented with 10% FBS. Following overnight incubation, the inserts were carefully removed, and the confluent monolayers were washed with PBS. Cells were then treated with sub-antiproliferative concentrations (1.0, 2.5 and 5.0 µM) of compound **4a** in the EMEM medium containing 2% FBS. Images were then captured at 0, 24, and 48 h post-treatment using a Nikon Eclipse TS100 fluorescence microscope (Nikon Instruments Europe, Amstelveen, The Netherlands). Untreated cells served as the control, and the rate of wound closure (the decrease in size of the gaps devoid of cells) was measured using ImageJ software v1.54 (National Institutes of Health, Bethesda, MD, USA).

### 2.8. Boyden Chamber Assay

Compound **4a**’s capacity to inhibit tumour invasion was assessed using the Boyden chamber assay, a three-dimensional model, on a profoundly invasive HPV-18 positive cell line. The polyethylene terephthalate (PET) membrane (8 μm pore size) and the thin layer of the matrigel basement matrix in special Boyden chamber inserts (BioCoat™ Matrigel^®^ Invasion Chambers, Corning Inc., Corning, NY, USA) were prehydrated (2 h, serum-free EMEM) and placed onto a 24-well plate. The cell suspension (50,000 cells/insert in 500 μL) prepared in the serum-free EMEM with sub-antiproliferative concentrations (1.0, 2.5, and 5.0 μM) of **4a** was nested in the upper compartments. Untreated cells were used as controls, with the EMEM medium supplemented with 10% FBS, and served as a chemoattractant in the lower chambers. After 24 h of incubation, supernatants and non-invading cells were removed cautiously with a cotton swab. The cells were then washed with PBS twice, fixed with ice-cold 96% ethanol, and stained with 1% crystal violet dye for 30 min in the dark. Subsequently, 4 images per chamber were taken with a Nikon Eclipse TS100 fluorescence microscope (Nikon Instruments Europe, Amstelveen, The Netherlands), and the number of invading cells were enumerated using ImageJ software (National Institutes of Health, Bethesda, MD, USA). The invasion rate was determined by comparing the number of invaded cells in the treated samples with the number of invaded cells in the untreated control samples.

### 2.9. Molecular Simulation

Molecular mechanical level investigations were applied to elucidate how compound **4a** binds to the tubulin–microtubule system. The first replica exchange solute tempering (REST) dynamics [27,28] were performed for the original protein ligand crystal structure to sample the conformational space of the binding pocket. The 10 Å environment of the original ligand in the colchicine-binding site was selected, including amino acids and cofactors, and 100 ns-long REST running was performed with 4 replicas. The lowest trajectory was generated at 310 K, and the suggested default temperature values were accepted for higher energy replicas. Using the trajectory with the lowest temperature, ten random structures and the **4a** ligand docked to the colchicine binding pocket in all ten cases were selected using the Glide module of the Schrödinger suite [29]. During the docking, the XP protocol was applied with enhanced sampling ligand conformation generation, and the following steps were applied for each protein–ligand complex. A single-strand 150 ns-long molecular dynamics calculation was performed at 315 K. Then, having a more realistic binding free energy value, molecular mechanics with a generalised born and surface area solvation (MM/GBSA) method [30] was applied in all of the 10 complexes. The binding free energy for each trajectory was calculated using the thermal MMGBSA python script of the Schrödinger suite [31] for the 50–150 ns intervals, while the first 50 ns was handled as a relaxation period.

### 2.10. Estrogenic Activity Assay

The in vitro oestrogenic activity of our test compound was determined by utilizing the T47D-ERE-Luc^Neo^, an ERα-positive cell line, stably transfected with a luciferase reporter cassette [32]. On the first day of the experiment, cells were seeded onto 96-well microplates in a density of 50,000 cells/well. After 72 h of incubation, the medium was changed to phenol red-free RPMI, containing 10% charcoal-stripped FBS. This step was repeated for another four consecutive days to deplete the intracellular oestrogen content. On the ninth day, cells were treated with eight different concentrations of 17β-oestradiol (3 × 10^−14^–10^−10^ M), 2-methoxyestradiol (10^−12^–10^−5^ M, Merck Life Science Kft., Budapest, Hungary), or the test compound (10^−8^–3 × 10^−5^ M). After 24 h of incubation, the supernatant was removed from the wells and the One-Glo^™^ firefly luciferase reagent was added according to the manufacturer’s instructions (Promega Corp., Madison, WI, USA). Following a 5 min incubation period, the luciferase enzyme activity was measured using a luminometer (FLUOstar OPTIMA, BMG Labtech GmbH, Ortenberg, Germany). Each concentration was assayed in triplicates, repeated twice. Relative luminescence units (RLU) were normalised to percentage values, and sigmoidal concentration–response curves were fitted to the data points using GraphPad Prism 9.5.1 (GraphPad Software, San Diego, CA, USA).

### 2.11. MTT Assay of ***4a*** on the Growth Inhibition of MCF7 and T47D Cell Lines

The MTT assay was conducted as described in Section 2.5, with the incubation time extended to 72 h. IC_50_ values were determined by fitting sigmoidal concentration–response curves using GraphPad Prism 9.5.1 (GraphPad Software, San Diego, CA, USA). This was undertaken to ascertain the antiproliferative activity of compound **4a** on ER-α positive MCF7 and T47D breast cancer cell lines.

### 2.12. Statistical Analysis

Statistical analysis was performed using GraphPad Prism 5.01 (GraphPad Software, San Diego, CA, USA). One-way analysis of variance (ANOVA) followed by the Dunnett post-test was used to determine the statistical significance. All results shown represent means ± SEM from a minimum of two independent experiments with three replicates. *, **, and *** denote *p* < 0.05, *p* < 0.01, and *p* < 0.001, respectively, as compared to the control.

## 3. Results

### 3.1. Apoptosis-Inducing Effect of ***4a*** with Fluorescent Double Staining

**Figure 2:**
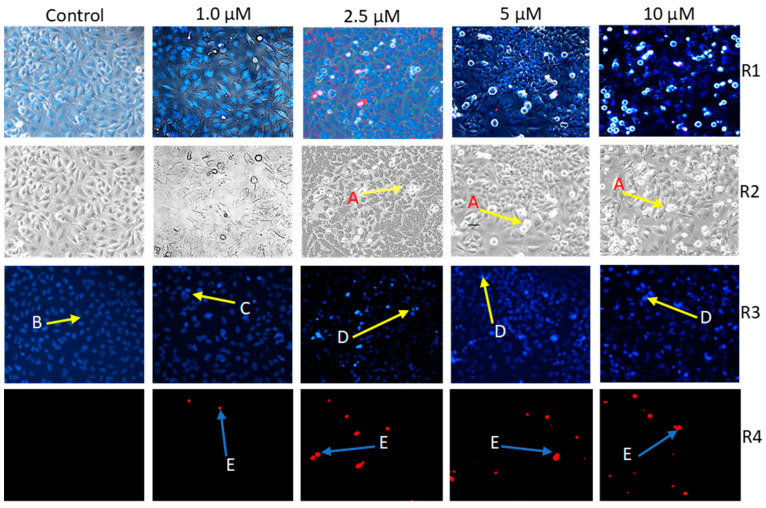
Representative photos of two independent HOPI experiments performed on three parallels. Compound **4a**-induced morphological changes on HeLa cells (**A**) under Brightfield (**R2** panel). Intact (**B**), early apoptotic (**C**), late apoptotic (**D**), and necrotic (**E**) HeLa nuclei stained with Hoechst 33258 (blue fluorescence, **R3** panel) and propidium iodide (red fluorescence, **R4** panel) after 24 h of treatment at four distinct concentrations. Panel **R1** represents a fusion of corresponding photos from R2, R3, and R4. Photos captured with fluorescent microscopy at 10× magnification.

When exposing HeLa cells to varying concentrations of compound **4a** for 48 h, noticeable changes were observed in both the morphological appearance of the cells and the integrity of their membranes. Fluorescence imaging conducted during this timeframe revealed a decrease in the number of viable cells, and the extent of this reduction directly correlated with the concentration of compound **4a**. Additionally, there was a noteworthy and concentration-dependent escalation in the number of nuclei emitting intense blue fluorescence, signifying the DNA condensation-a characteristic feature of early apoptosis. Furthermore, a discernible number of images exhibited red fluorescence, indicating the presence of secondary necrotic cells, and thereby implying potential damage to the cell membranes (Figure 2).

### 3.2. Compound ***4a*** Induced Cell Cycle Disturbance via Flow Cytometry

**Figure 3:**
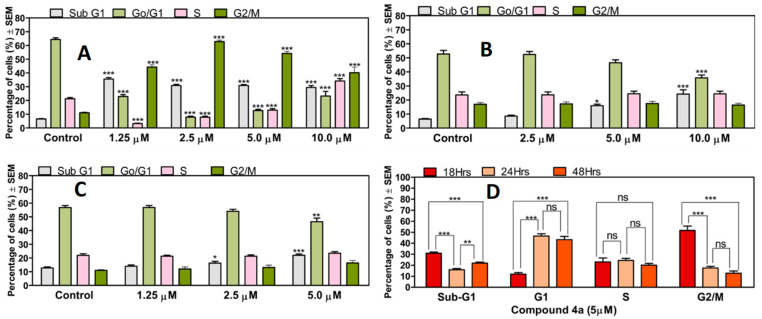
**4a**-induced cell cycle disturbances are marked with a significant increase in the sub-G1 cell population, accompanied with a cell blockade at the G2/M phase (**A**) observed 18 h post-treatment. Extended incubation for 24 (**B**) and 48 (**C**) h only resulted in significant rises in the hypodiploid sub-G1 population at 5, 10 µM and 2.5, 5 µM, respectively. Statistical analysis conducted to assess the impact of the incubation time on cell cycle progression in HeLa cells treated with **4a** at a concentration of 5 µM (**D**). Results are expressed as mean values ± SEM of the data on three separate measurements with triplicates, ns: no significance, *, **, and *** indicate *p* < 0.05, 0.01, and *p* < 0.001, respectively, as compared to untreated control samples.

The impact of compound **4a** on cell cycle progression was investigated in vitro at three different time intervals (18, 24, and 48 h) though the analysis of cellular DNA content using PI. After treating HeLa cells with compound **4a**, and the subsequent incubation for 18 h, a swift and significant cell arrest at the G2/M phase, along with a noteworthy significant rise in the hypodiploid sub-G1 population, was observed. This effect was consistent across all tested concentrations (1.25, 2.5, 5.0, and 10 µM). Longer incubations of 24 and 48 h led to a concentration-based significant increase only in the sub-G1 population, and not the G2/M phase, at the highest two concentrations. The statistical analysis of the impact of incubation time on cell cycle progression in HeLa cells treated with **4a** at a concentration of 5 µM (corresponding to its IC_50_ value as previously published, [19]) revealed notable findings. The results indicated a significant increase in the sub-G1 phase at 18 and 48 h when compared to 24 h. Moreover, a significant decrease in the G1 cell population was evident at 18 h when compared with 24 and 48 h. However, no significant differences were observed in the cell population in the S-phase across all tested time intervals. Nonetheless, a significant increase in the G2/M population at 18 h was observed, relative to both the 24 and 48 h time intervals (Figure 3).

### 3.3. Compound ***4a*** Lacked Swift Cytotoxic Effects

**Figure 4:**
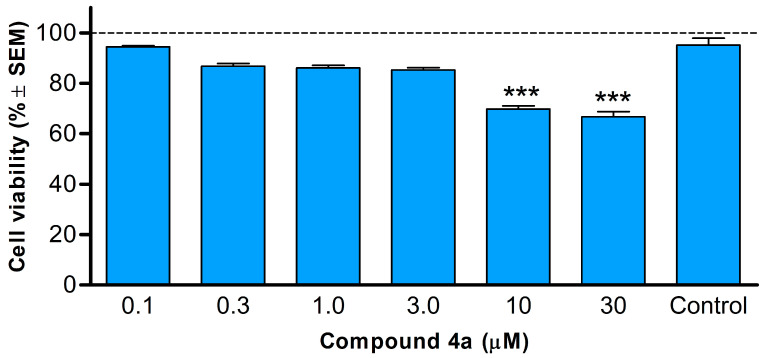
Cell viability of HeLa cells following 24 h incubation with **4a**. Data include two independent experiments conducted with five parallels. Control: untreated cells. Results are expressed as cell viability % ± SEM. *** denote *p* < 0.001. Non-significant changes are not indicated.

A study was undertaken to examine cell growth inhibitory effects of **4a** on HeLa cells, with the goal of determining whether **4a** induced rapid cytotoxicity following a 24 h incubation period. This validation was critical as previous findings on cell cycle progression, conducted after an 18 h incubation of HeLa cells with **4a**, had shown the induction of apoptosis via a significant increase in the sub-G1 population. The MTT assay results for compound **4a** following a 24 h incubation period with HeLa cells revealed diminished inhibition in cell growth. Notably, even at concentrations corresponding the IC_50_ of **4a,** there was no significant alteration in cell viability (Figure 4).

### 3.4. Effects of Compound ***4a*** on Tubulin Polymerisation

**Figure 5:**
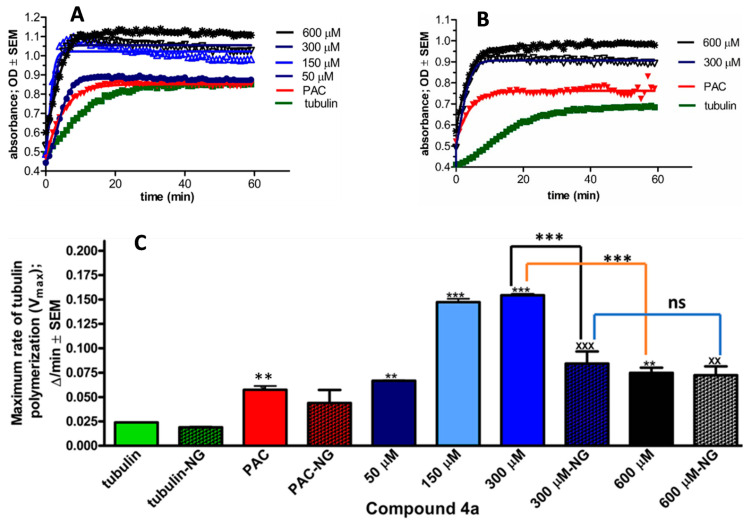
The direct effect of **4a** on tubulin polymerisation in a cell-independent experimental system in vitro. Kinetic curves of 60 min kinetic reaction, after incubation with **4a** at 50, 150, 300, and 600 μM. General tubulin buffer and 10 μM paclitaxel (PAC) were used as negative and positive controls, respectively. Kinetic curve (**A**) in the presence of glycerol, while (**B**) with no glycerol. Maximum rate of tubulin polymerisation (Vmax) in the presence and absence of glycerol (**C**). Results are expressed as means ± SEM of the data on two separate measurements with duplicates. ns indicates *p* > 0.05, **, ^xx^ indicates *p* < 0.01, and ***, ^xxx^ indicates *p* < 0.001, relative to the corresponding negative control samples. NG (no glycerol).

Based on the observed G2/M phase blockade from compound **4a**, we sought to assess its influence on microtubule dynamics. A cell-independent tubulin polymerisation assay was carried out in both the presence and absence of glycerol. Following the manufacturer’s guidelines and taking into account the IC_50_ value of **4a**, concentrations of (50, 150, 300, and 600 µM) of the test compound were examined. In this study, paclitaxel was utilised as a positive control. The findings revealed a concentration-dependent augmentation in both the rate (Vmax) and extent of tubulin polymerisation, attaining a maximum rate of 300 µM in both the presence and absence of glycerol (Figure 5). Beyond this threshold, in the presence of glycerol, there was a significant decrease in the maximum rate of tubulin polymerisation. Conversely, in the absence of glycerol, there was no significant difference observed in the maximum rate of tubulin polymerisation between 300 µM and 600 µM. Remarkably, at all tested concentrations of **4a**, there was a significant increase in tubulin polymerisation relative to the control, regardless of the presence or absence of glycerol.

### 3.5. Inhibitory Effect of ***4a*** on HeLa Cell Migration

**Figure 6:**
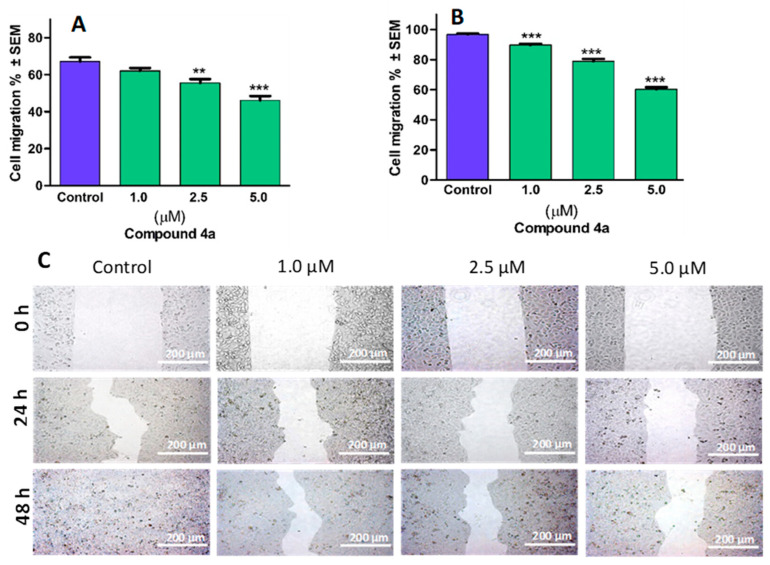
Compound **4a** inhibited the migration of cervical cancer cells (HeLa). Quantified rates of wound closure were significantly diminished in the presence of compound **4a.** Graphs indicate the percentage of cell migration at 24 h (**A**) and 48 h (**B**) post-treatment of HeLa cells, relative to the control. Representative images of reduced wound healing at 0, 24, and 48 h post-treatment (**C**). Results are expressed as mean values ± SEM of the data on two separate measurements with triplicates. ** and *** indicate *p* < 0.01 and *p* < 0.001, respectively, compared to control.

To investigate compound **4a**’s antimetastatic properties, the evaluation of changes in the cellular migratory capability in response to the sub-antiproliferative concentrations of **4a** was performed. Comparing alterations observed at 24 h and 48 h post-treatment with untreated controls revealed that compound **4a** demonstrates an antimigratory effect, dependent on both the concentration and duration (Figure 6). Further validation through image analysis confirmed a significant reduction in the migratory capacity of HeLa cells—measuring 53.9% and 39.6%, respectively—after 24 or 48 h of treatment with 5.0 µM of the compound relative to the control. 

### 3.6. Tumour Anti-Invasive Activity of ***4a*** on HeLa Cells

**Figure 7:**
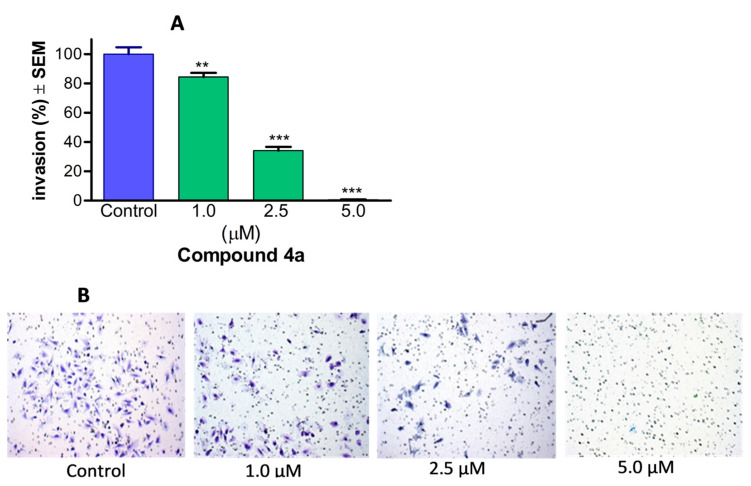
Compound **4a** markedly diminished the invasiveness of cervical cancer cells (HeLa). The anti-invasive potential of the test compound is illustrated by representative images (**B**), and is quantified by the percentage of invading cells in the Boyden chamber containing different concentrations of **4a**, using an EMEM medium supplemented with 10% FBS as a chemoattractant (**A**). Results are presented as mean values ± SEM of the data from three separate measurements with duplicates. ** and *** indicate *p* < 0.01 and *p* < 0.001, respectively, compared to untreated control samples.

To assess the invasion of cancer cells, Boyden chamber inserts containing a matrigel membrane were employed. Image-based data were gathered for each sample, enabling the calculation of the invading cells per well. This count was then utilised to determine and express the percentage of cancer cells that successfully infiltrated the surrounding area. When compared to control samples, it was observed that **4a** demonstrated a significant and concentration-dependent inhibitory effect on cancer cell invasion when tested within a concentration range from 1.0 to 5.0 µM (Figure 7). Notably, when exposed to a concentration of 5.0 µM for 24 h, the number of invading HPV-18 positive cervical cancer cells decreased by more than 95%. These findings serve to validate the anti-invasive potential of **4a,** even at concentrations below its antiproliferative threshold.

### 3.7. Molecular Simulation of ***4a*** Binding in Tubulin

The molecular docking assay predicts and evaluates the binding profile of a compound with the tubulin heterodimer, a critical component of microtubules. Based on our results of tubulin polymerisation, we planned to investigate the interaction of compound **4a** with well-established binding sites within a tubulin heterodimer, a target for the therapeutically utilised MTAs.

**Figure 8:**
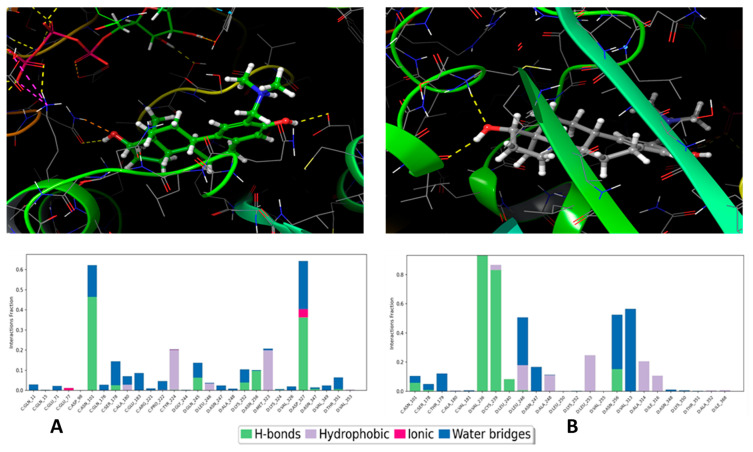
Representative binding poses of **4a,** and the best two binding pocket models (**top**) and its important residual interactions (**bottom**) of the same models in the 150 ns-long MD simulations. The left (**A**) and right (**B**) panels represent Rec01 and Rec05 cases, respectively. The yellow dashed lines in the pictures represent the H-bond interactions.

The binding free energy of ten ensembles were determined with molecular mechanics with generalised born and surface area solvation (MM/GBSA) calculation methods to sample the binding possibilities of the **4a** ligand in the colchicine-binding site, and the calculation results are presented in Table 1. According to Table 1, two cases showed strong binding abilities, and the representative ligand geometries are presented in Figure 8. To explore the possible principal interactions between the ligand and protein, the Simulation Interaction Diagram package of the Schrodinger suite was applied. In the two strongest binding cases (Rec01 and Rec05 in Figure 8A and Figure 8B, respectively), two characteristic interactions were identified (Rec01: ASN101, ASP327; Rec05: VAL236, CYS239). These interactions persisted most of the time during the simulation (60% and 80%, respectively), and, according to the ligand RMSD values, these ligand geometries show stable binding poses. We would like to mention that, in all of the 10 cases, the protein–ligand interactions were strong enough to hold the **4a** molecule in the colchicine binding pocket. These results show that the A-ring modified steroids, **4a**, can form stable connections to the colchicine binding pocket. Finally, we would like to note that the Taxol-binding site was also investigated as a further possible ligand binding pocket for molecules with a sterane skeleton. During the 150 ns-long single-strand simulation, the ligand detached from the binding pocket, which suggested a less favourable binding capacity. 

### 3.8. Estrogenic Activity of Compound ***4a***

**Figure 9:**
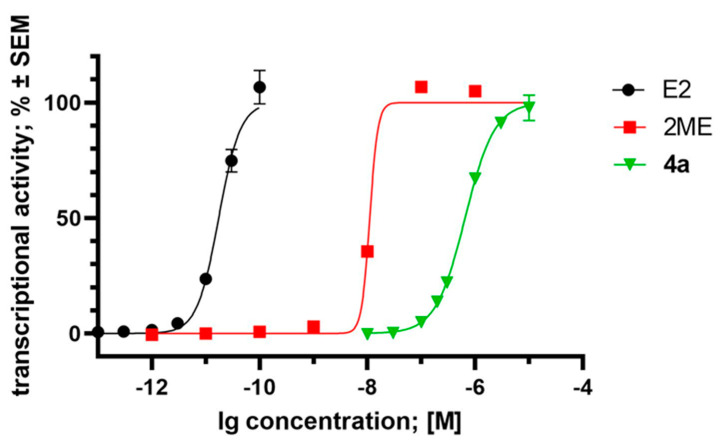
Oestrogenic effect of compound **4a**, 2-methoxyestradiol (2ME), and 17β-oestradiol (E2), expressed as the transcriptional activity of the oestrogen-responsive luciferase reporter cassette transfected into the T47D breast cancer cell line, T47D-ERE-Luc^Neo^. Data are based on two independent experiments performed in triplicate, and are expressed as average ± SEM.

Since compound **4a** is structurally closely related to 2ME and they can be considered as analogs of 17β-oestradiol, their possible oestrogenic activities may contribute to their pharmacological features. Our experiment, aimed at determining the transcriptional activity of the oestrogen-dependent luciferase gene transfected into T47D breast cancer cells, yielded notable results. It can be observed that compound **4a** (EC_50_ = 6.53 × 10^−7^ M) and 2ME (EC_50_ = 1.11 × 10^−8^ M) exhibit oestrogenic activity at least three orders of magnitude weaker than the positive control, endogenous 17β-oestradiol (EC_50_ = 1.74 × 10^−11^ M) (Figure 9). This finding aligns well with previously published data, particularly regarding 2ME [33]. On the other hand, compound **4a**, similarly to 2ME, displays its antiproliferative, antimetastatic, and hormonal activity in the same concentration range.

### 3.9. Antiproliferative Activity of ***4a*** on T47D and MCF7 Breast Cancer Cell Lines

Comparatively, **4a** showcased a diminished growth inhibitory effect on the T47D and MCF7 breast cancer cell lines relative to the cervical cancer cell line, HeLa. Notably, compound **4a** exhibited an IC_50_ value of 4.53 μM on HeLa cells [19], while, on T47D and MCF7, IC_50_ values of 16.92 and 19.18 μM were observed, respectively.

## 4. Discussion

On a global scale, it is anticipated that there will be 34 million new cancer cases in the year 2070, which is double the estimated number in 2018 [34]. Cervical cancer is the fourth most common cancer in women worldwide, responsible for 20% of cancer-related deaths [3]. Consequently, there is a high demand for more effective and better-tolerated drugs in order to address this specific type of cancer. Our previous study highlighted the growth inhibitory effects of compound **4a** on selected gynaecological cancer cell lines. Furthermore, we demonstrated its exceptional tumour selectivity [19], addressing a significant challenge faced by new anticancer drug candidates [35]. This paper aims to provide compelling evidence regarding the mechanism of action responsible for the observed antiproliferative effect of compound **4a**. 

Numerous research studies have provided evidence showcasing the diverse antitumour properties of 2ME. Its mechanism of action encompasses a range of effects, including antiproliferative, antitubulin, antiangiogenic, antimetastatic, and proapoptotic activities [36,37]. In light of the structural homology existing between compound **4a** and 2ME, our objective was to elucidate the mechanism underlying the antiproliferative activity exhibited by compound **4a** on HeLa cells. 

Based on the data linked to common apoptosis markers, compound **4a** emerges as a promising antiproliferative agent capable of inducing apoptosis. This insight stems from a comprehensive HO/PI double staining analysis. The changes in the cell morphology hinted at apoptosis induced by **4a**, with a notable rise in cells exhibiting condensed DNA post-treatment, reinforcing this observation. Additionally, the intense blue fluorescence on the HO panel signified nuclear condensation, indicative of an early stage in programmed cell death [38]. The 48 h incubation period may have caused certain early apoptotic cells to progress into late-apoptotic or secondary necrotic stages. This transition is indicated by the red fluorescence emitted by some cells when stained with PI.

The flow cytometry analysis further confirmed the apoptosis-inducing effect of **4a**, and provided additional insights into certain aspects regarding the mode of action of compound **4a**. Specifically, treated cells exhibited rapid arrest at the G2/M phase, accompanied by a significant increase in the hypodiploid sub-G1 population after 18 h of incubation. However, extended incubation periods of 24 and 48 h resulted only in a significant increase in the sub-G1 population, with no notable change observed in the G2/M population. The significant and noteworthy increase in the sub-G1 cell population observed upon the incubation of the HeLa cells with **4a** for 18 and 48 h, coupled with a notable rise in the G2/M phase after only 18 h of treatment with **4a**, may be partially attributed to a phenomenon associated with certain potent antimitotic agents. Antimitotic compounds are known to activate the spindle assembly checkpoint (SAC), resulting in mitotic arrest [39]. After an extended period of arrest, various outcomes have been observed. Some cells undergo cell death during mitosis, while others exit mitosis without division and then re-enter the interphase [40,41]. Upon returning to the interphase, certain cell lines experience cell-cycle arrest, some undergo cell death (apoptosis), and others replicate their genomes again. This may therefore partly explain the observed significant rise in the sub-G1 population at 18 and 48 h, with the absence of G2/M cell arrest at the extended time (24 and 48 h) of incubation. 

The MTT assay was conducted to evaluate how **4a** affected the growth of HeLa cells after 24 h of incubation. This was important in determining whether the sharp rise in the sub-G1 cell population observed during the 18 h cell cycle analysis was mainly caused by apoptosis or was a direct cytotoxic impact of **4a**. The results showed diminished cell growth inhibition, with no significant change in cell viability at a concentration corresponding to the IC_50_ value of **4a**. Therefore, the significant increase in the sub-G1 population after 18 h of incubation is likely due to induction apoptosis rather than a direct cytotoxic effect of **4a**.

Tubulin polymerisation plays a vital role in mitosis. Importantly, 2ME has been documented as a suppressor of tubulin polymerisation via targeting the colchicine-binding site on tubulin [42]. Conversely, certain steroid analogs exhibit the ability to augment the assembly of microtubules from tubulin dimers [43]. Similarly, we investigated the direct influence of compound **4a** on tubulin polymerisation in vitro. We utilised a cell-independent system, specifically optimised to evaluate test compounds for their potential to either enhance or inhibit tubulin polymerisation. This system includes glycerol in a carefully controlled concentration to expedite tubulin polymerisation. Consequently, all recorded absorbance values represent the combined effect of glycerol and the test compound. Compound **4a** significantly enhanced tubulin polymerisation at all tested concentrations. However, there was a noticeable decrease in the maximum rate of tubulin polymerisation between the concentrations of 300 µM and 600 µM. As glycerol is known to promote tubulin polymerisation [44], we chose to further investigate the direct impact of **4a** in its absence. Compound **4a** exhibited its maximum tubulin polymerisation effect at approximately 300 µM. Beyond this concentration, at 600 µM, there was no significant alteration in the maximum rate of tubulin polymerisation. These values portray a genuine intrinsic effect of our test compound. When comparing the maximum rate of tubulin polymerisation induced by compound **4a** with and without glycerol, similar values are obtained, indicating the full binding of tubulin proteins, even with glycerol present. Since there is no notable difference in the effect of compound **4a** at 300 µM and 600 µM concentrations on the tubulin polymerisation rates in experiments without glycerol, 300 µM can be considered the optimal concentration for saturating tubulin-binding sites with **4a**.

Multiple epidemiological and clinical investigations have revealed that a notable percentage of fatalities associated with cancer can be ascribed to the emergence of metastases. Thus, inhibiting cell migration becomes a valuable target for therapeutic intervention, as it can prevent invasion and metastasis, ultimately leading to reduced mortality among patients [45]. Microtubules, composed of α/β-tubulin monomers, play a critical role in regulating cell shape, motility, transport, and division [46]. They undergo a dynamic instability, wherein they continually grow until reaching a critical point, at which they rapidly regress. This turnover process can influence the activity of actin fibre regulators like the Rho-guanine nucleotide exchange factor [47], which forms the delivery channels for membrane proteins required for focal adhesion assembly [48]. However, disrupting this turnover may impact the ability of microtubules to engage with focal adhesions, subsequently altering focal adhesion signalling and turnover, resulting in changes in cell migration [49]. Certain synthetic compounds, such as 2ME, demonstrate both antimitotic properties [50] and cell antimigratory activity in vitro [51], indicating their potential as anti-cancer agents. In our in vitro experimental assay, the drug candidate **4a** demonstrated a remarkable ability to inhibit the migration of highly invasive HeLa cells [52], in a manner that was dependent on both time and concentration. Notably, even at sub-antiproliferative concentrations in the micromolar range, a substantial inhibitory effect persisted, even after 48 h of incubation. The migration of cells necessitates their entry into the circulatory system though either intravasation into the lymphatic system or blood vessels. This process involves the interaction of cancer cells or cell aggregates with the endothelial barrier, leading to the induction of gap formation via diverse biochemical reactions and signal transduction pathways [53].

In addition to cell migration, the invasion and infiltration of surrounding tissues are pivotal for the development of tumour metastases. Consequently, we supplemented our wound-healing assay for cellular migration by incorporating a specialised three-dimensional cell invasion Boyden chamber assay. This assay aimed to mimic the extracellular environment of primary tumours. Our experimental findings revealed a concentration-dependent inhibition of cell invasion, induced by compound **4a**, achieving a maximum anti-invasive activity of over 95% at a concentration of 5.0 µM when compared to the control. These results were consistent with those observed in a study involving 2ME [54].

The mode of action of 2ME involves the interaction with the colchicine binding domain on β-tubulin, influencing microtubule dynamics [55]. Molecular mechanical calculations revealed **4a**’s affinity for the colchicine-binding site, similar to 2ME. However, while 2ME inhibits tubulin polymerisation, compound **4a** was found to actually enhance this process. Similar occurrences have been reported with various other structurally different compounds. For instance, cevipabulin, an analog of combretastatin [56], demonstrated distinctive antimitotic behaviour by binding to the vinca binding-site and boosting tubulin polymerisation [57]. This stands in contrast to the behaviour of known antimitotic agents, which typically inhibit tubulin polymerisation by binding to either the vinca or colchicine-binding sites on the tubulin [58].

Computational simulation provided two noteworthy binding poses in the colchicine binding pocket; however, orientations of **4a** in the binding pocket were quite different. Therefore, we cannot deduce that computation simulations suggest a unique binding pose. According to the computational investigations, four residues were responsible for the two binding poses. Interestingly, the modified part of the steroid did not participate directly in these bindings. Therefore, the modification is thought to have an orientation role in protein–ligand complex formation. More specifically, interaction diagrams of the strongest protein-binding ligand (Rec01 and Rec05) show characteristic hydrogen bonds between the -OH groups of the ligand and the ASN101 and ASP327 amino acids, VAL236, and CYS239 (Figure 8). Furthermore, the ligand exhibits less fluctuating in the case of the fifth receptor model as a result of assuming a more penetrative position in chain D of β-tubulin. Collectively, these findings reinforce our earlier results, which demonstrated the interaction of compound **4a** with tubulin and the consequent enhancement of tubulin polymerisation. Thus, the delicate balance between microtubule polymerisation and depolymerisation plays a crucial role in ceasing cell division [59]. Consequently, this directional shift is harnessed in tumour therapy, where agents, such as the depolymerizing agent nocodazole or the tubulin-polymerizing agent paclitaxel, act.

The stimulatory effect of 17β-oestradiol on the proliferation of malignant tissues and cells has been demonstrated through its binding to and activation of ERα. This activation results in the dissemination of primary tumour cells to distant organs. While our findings reveal that compound **4a** demonstrates significant oestrogenic activity, as determined through the luciferase reporter assay system using ERα transfected into the T47D breast cancer cell line, it also exhibits the notable inhibition of proliferation and cell motility in HeLa cells. In other studies, the absence of the ERα protein in various cervical cancer cell lines, including HeLa cells has been reported [60].

Additionally, they uncovered that HeLa cells express protein, a variant of ERα which shares similarities with ERα in terms of DNA and ligand-binding domains, albeit lacking the activator function-1 (AF-1) region found in ERα [61]. However, despite the similarities in their ligand-binding domain, compound **4a** seemed to have a diverse interaction with the signalling pathways. Notably, compound **4a** exhibited a higher growth inhibitory effect on HeLa cells, expressing ERα-36, than on T47D and MCF7 breast cancer cell lines, where it expressed the native form of ERα. In addition to directly interacting with the colchicine-binding site on the tubulin protein, as demonstrated with our computational simulation, compound **4a**’s effect on the signal transduction pathway of ERα-36 may contribute to its observed antiproliferative and cell motility inhibiting effects on HeLa cells. Thus, further investigations are necessary to thoroughly elucidate the interaction between compound **4a** and the cervical ERα-36 receptor.

## 5. Conclusions

Owing to its impressive antiproliferative activity and tumour selectivity, compound **4a** was investigated for its mechanism of action on the most susceptible cell line HeLa. Compound **4a** exhibited significant antimetastatic and anti-invasive effects, along with the G2/M cell cycle blockade on HeLa cells. Additionally, **4a** showed an affinity for binding to and enhancing tubulin polymerisation, which partially explains its observed antiproliferative and antimetastatic activity. However, **4a** also demonstrated oestrogenic activity through the activation of ERα. Since ERα-36, a variant of ERα, is the predominant form in cervical cancer tissues, further research is needed to fully comprehend the effects of the interaction between **4a** and ERα-36. This interaction might potentially provide additional insight into the observed anticancer properties of **4a** on cervical tumours. Ultimately, our study highlights the potential of oestradiol derivatives with A-ring modifications as promising candidates for the development of novel anticancer agents targeting cervical cancer.

## Figures and Tables

**Table 1:** MM/GBSA binding free energy values (kcal/mol) of **4a** in the colchicine binding pocket for the generated receptor models. Results with relaxation means that the separated ligand and protein environment were minimised.

Receptor Models	MM/GBSA Binding Free Energy (No Relaxation)		MM/GBSA Binding Free Energy (with Relaxation)	
	(Average kcal/mol)	Standard Deviation	(Average kcal/mol)	Standard Deviation
Rec01	−41.9337	5.44	−44.0776	5.54
Rec02	−22.8293	5.25	−25.8875	5.86
Rec03	−20.8504	3.93	−23.6403	3.96
Rec04	−18.4347	5.35	−21.4334	6.09
Rec05	−40.4151	3.98	−43.1558	4.00
Rec06	−29.5527	3.57	−33.3576	3.57
Rec07	−9.6403	5.92	−12.0252	5.86
Rec08	−15.255	4.91	−17.5359	4.67
Rec09	−7.1579	5.85	−9.5942	5.83
Rec10	−16.8539	4.87	−18.4593	5.45

## Data Availability

Data will be available upon request.
